# Aumolertinib Effectively Reduces Clinical Symptoms of an EGFR L858R-Mutant Non-Small Cell Lung Cancer Case Coupled With Osimertinib-Induced Cardiotoxicity: Case Report and Review

**DOI:** 10.3389/fendo.2022.833929

**Published:** 2022-05-23

**Authors:** Qianqian Zhang, Haiyang Liu, Jia Yang

**Affiliations:** Department of Respiratory and Critical Care Medicine, Henan Provincial People’s Hospital, Zhengzhou, China

**Keywords:** aumolertinib, L858R, osimertinib, cardiac failure, NSCLC

## Abstract

Osimertinib, a third-generation epidermal growth factor receptor tyrosine kinase inhibitor (EGFR-TKI) first-line therapy, has shown good clinical outcomes in non-small cell lung cancer (NSCLC), but some serious adverse events such as cardiotoxicity have also been reported. Here, we present the first NSCLC case with osimertinib-induced cardiac failure. The case is successfully being treated by switching to another third-generation TKI, aumolertinib. A 62-year-old non-smoking woman was initially diagnosed with stage cT2aN2M1c IVB NSCLC with synchronous brain and bone metastasis in April 2020. Further genetic screening of the patient identified Leu858Arg (L858R) mutation in EGFR; thus, the patient was administered third-generation TKI osimertinib (80 mg/day) for 6 months. This treatment with osimertinib led to serious cardiac failure but no significant reduction in NSCLC tumor size. To cope with these conditions, another third-generation TKI, aumolertinib (110 mg/day), along with a supplement treatment plan was prescribed to the patient. Interestingly, this new treatment plan of aumolertinib significantly inhibited tumor growth in 8 months. Therefore, we conclude that the administration of second-line aumolertinib 110 mg/day has fewer adverse reactions and high efficacy against NSCLC as compared to osimertinib therapy.

## Introduction

Lung cancer is one of the leading causes of cancer-related death worldwide, including China (1). Lung cancer can be divided into small cell lung cancer (SCLC) and non-small cell lung cancer (NSCLC) according to cell morphology, and approximately 85% of cancers are NSCLC. It has been well established that the epidermal growth factor receptor (EGFR) is a common driver gene for NSCLC, and EGFR mutations can be detected in about 40%–60% of the East Asian population (2). In the last decades, the treatment options for NSCLC have improved dramatically and standard platinum doublet chemotherapy has been substituted by tyrosine kinase inhibitors (TKIs). More recently, TKIs have become the standard treatment for the EGFR-mutant for NSCLC, when patients are diagnosed with pathogenic mutations. The most common cluster of mutations in *EGFR* includes deletions around the LeuArgGluAla motif (residues 746–750) of exon 19, and among all the Leu858Arg (L858R) point mutations in exon 21, each accounting for 45% of all EGFR mutations (3, 4).

The presence of these mutations in NSCLC patients showed 9–14 months of progression-free survival (PFS) from those who positively respond to the first- and second-generation TKI (5–7). Nevertheless, about 60% of the patients with the EGFR T790M mutation who initially respond positively to the EGFR inhibitors such as gefitinib, erlotinib, afatinib, and dacomitinib in NSCLC eventually develop acquired drug resistance (ADR) in 9–15 months (8, 9). Third-generation EGFR-TKIs, such as osimertinib and aumolertinib, can minimize ADR. Based on the positive results obtained from clinical trials, the Food and Drug Administration (FDA) approved osimertinib for the treatment of patients with metastatic T790M- and EGFR-positive mutation in NSCLC (7, 10). In the FLAURA clinical trial, osimertinib showed the list of adverse events (AEs), and the most reported AEs were rash or acne, diarrhea, and dry skin. Among potentially life-threatening adverse reactions, cardiac failure was reported in 12 patients (4%) in the osimertinib group. The significant characteristics of the patients in this category were ejection fraction decrease, and electrocardiogram QT prolongation was reported in 28 patients (10%). In addition, interstitial lung disease (ILD) was also reported in 11 patients (4%) from the osimertinib treatment group (10). To date, no effective and alternative therapy has been recommended to fight the side effects of the osimertinib. Here, in this short report, we present a female case diagnosed with advanced NSCLC with L858R mutation in EGFR, which has presented grade 3 cardiac failure after being treated with osimertinib for 6 months; we successfully relieved the symptoms after switching the treatment with alternative third-generation EGFR-TKI aumolertinib.

## Case Presentation

**Figure 1:**
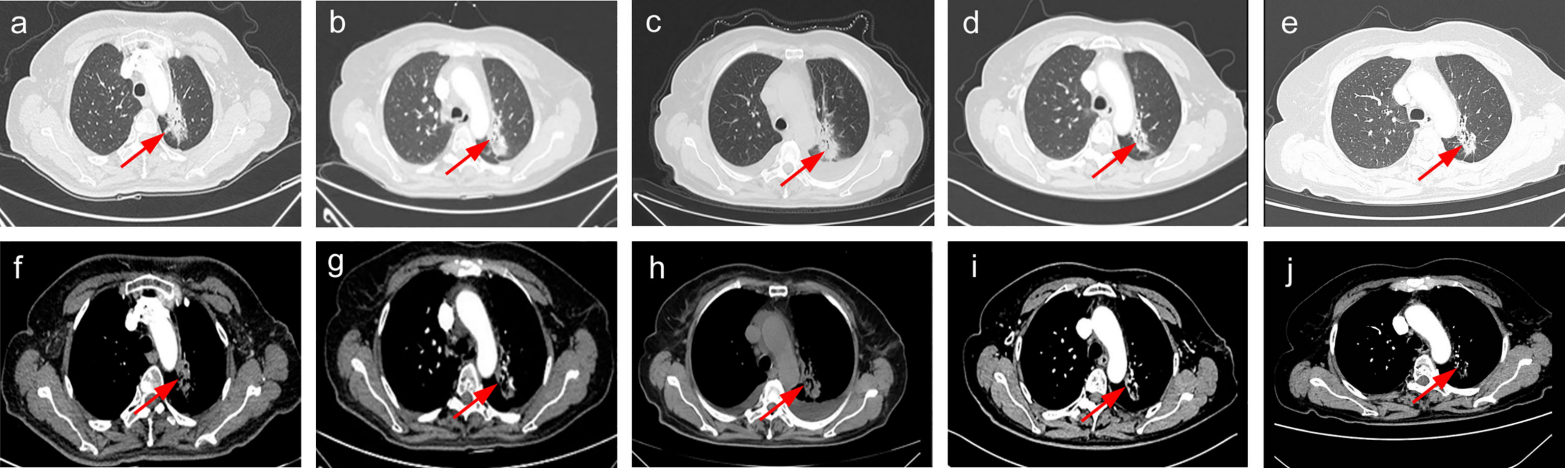
The clinical course according to check CT scan findings. **(A, F)** Baseline CT scan at diagnosis in April 2020. **(B, G)** SD on osimertinib in July 2020. **(C, H)** SD on osimertinib in October 2020. **(D, I)** PR on aumolertinib in March 2021. **(E, J)** SD on aumolertinib in July 2021. PR, partial response. SD, stable disease.

A 62-year-old non-smoker Chinese woman consulted Henan Provincial People’s Hospital with a complaint of mental illness in April 2020. Her family also complained chest congestion and discomfort. To diagnose the illness, the chest computed tomography (CT) scan was performed, which revealed a mass (3.9 × 2.7cm) in the left lung (**Figures 1A, F**), and a subsequent bronchoscopy revealed an NSCLC with mediastinal enlarged lymph nodes. Furthermore, the magnetic resonance imaging (MRI) scans revealed multiple metastases in the brain region in the form of nodular enhancement. In addition, chest-enhanced CT scan disclosed multiple abnormal density shadows that were considered as bone multiple metastases (data not shown).

**Figure 2:**
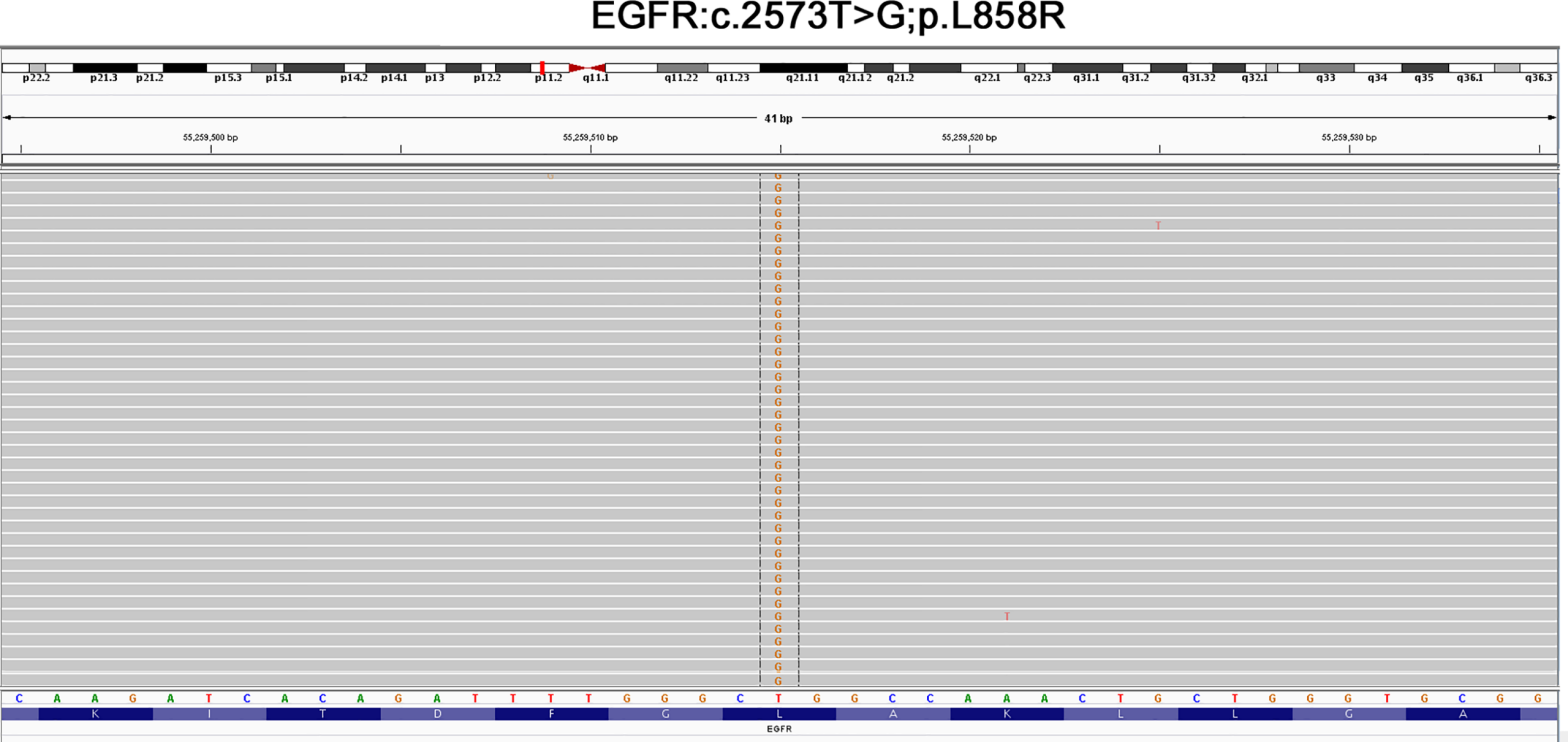
The next-generation sequencing analysis revealed that L858R mutation in exon 21 of the EGFR gene was visualized by IGV software.

Additionally, clinical serological laboratory testing showed elevated levels of carcinoma embryonic antigen (CEA) and neuron-specific enolase (NSE) (**Table 1**), and the immunohistochemistry analysis showed adenocarcinoma. The patient’s liver and kidney were observed as normal, and the normal heart function was shown by the marker of cardiac function [N-terminal brain natriuretic peptide precursor (NT-proBNP)] with a value of 70 ng/L (normal range, <300 ng/L). According to the American Joint Committee on Cancer (AJCC) version 8.0 for the TNM staging system, the current diagnosis of the patient revealed a presence of NSCLC adenocarcinoma at the cT2aN2M1c IVB clinical stage (including brain and bone metastasis). Moreover, the next-generation sequencing analysis discovered L858R mutation in exon 21 of the EGFR gene (**Figure 2**). Following complete diagnosis, we have requested a clinical history of the patient and her family and found that her family has no history of mental disease or cancer.

**Table 1:** The serological examination results at cardiac failure and during recovery treatment.

Time	Serological Markers			
	CEA (ng/ml)	NSE (ng/ml)	SCC (ng/ml)	CYFRA21 (ng/ml)
2020.04	60	13.51	0.8	4.89
2020.10	5.98	7.20	0.8	3.67
2020.11	7.69	8.56	0.9	3.84
2021.03	9.94	7.08	0.6	3.52
2021.07	9.73	6.65	0.8	3.66

**Figure 3:**
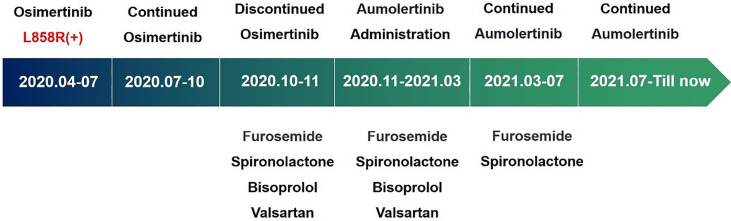
The medicine administration with indicated time periods shown as a flowchart diagram.

**Figure 4:**
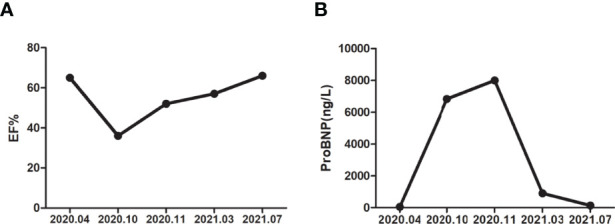
The detection of cardiac function in different time periods. **(A)** Ejection fraction in different time periods. **(B)** Brain natriuretic peptide precursor (pro-BNP) in different time periods.

**Figure 5:**
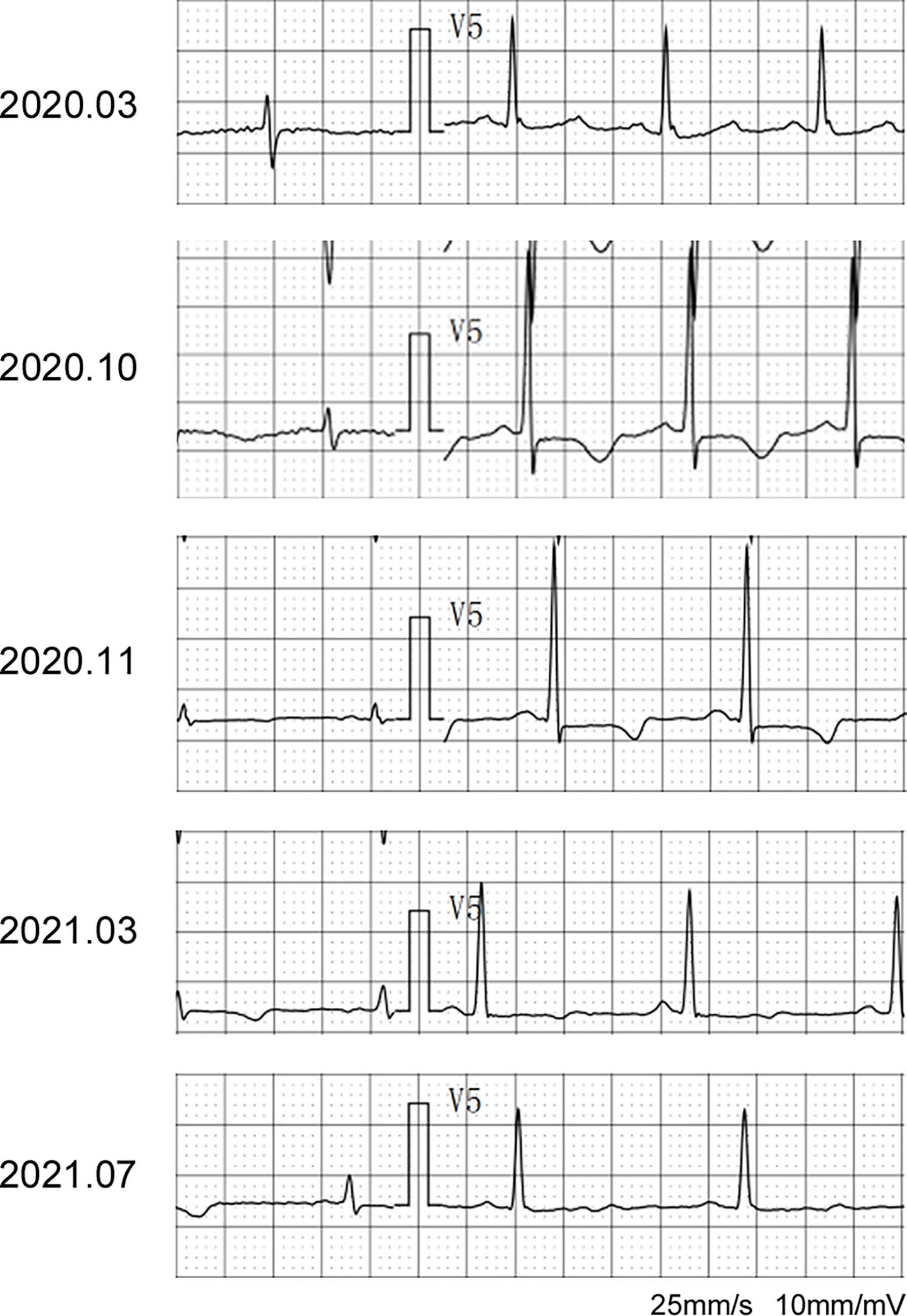
The changes in RV5 of electrocardiogram at cardiac failure and during recovery treatment.

The patient was asked to sign a consent form for the administration of the anti-tumor therapy. Written and verbal explanations about benefits and adverse reactions were given to the patient and attending persons. Firstly, the patient was prescribed osimertinib (80 mg po qd) and ibandronate (4 mg) for 6 months, with routine checkups. The entire treatment timeline of anti-tumor therapy is shown in a flowchart diagram (**Figure 3**). The follow-up of the first 3 months showed a stable tumor in the lung nidus (3.9 × 2.6cm) (**Figures 1B, G**). However, 6 months later, she complained of physical weakness and lower limb edema. To explore the symptoms and to dig out the causes of these complaints, chest CT, MRI, ECG, and serological analysis were performed. The results of the CT revealed no significant change in tumor size (3.9 × 2.6 cm) (**Figures 1C, H**). However, an MRI of the heart exposed pleural and pericardial effusions, and the echocardiogram (ECG) further indicated an obvious severe hypokinesis, with a left ventricular ejection fraction (LVEF) of 36% and the left ventricle was dysfunctioning (**Figure 4A**; **Tables 2** and **3**). In addition, the level of NT pro-BNP was gravely increased (6,830  ng/L) (**Figure 4B**). Considering that the patient had no history of cardiac disease and hypertension, we suspected that osimertinib may induce cardiotoxicity with CTCAE grade 3. This prompted us to discontinue osimertinib. Next, we prescribed the new treatment plan with furosemide (20 mg bid), spironolactone (20 mg bid), bisoprolol (20 mg qd), and valsartan (50 mg bid) to the patient to improve cardiac function and physical condition. Four weeks after the new treatment regimen, the LVEF improved to 52% (**Figure 4A**) including better cardiac function (**Table 3**); however, NT pro-BNP was further welled to 8,000 ng/L (**Figure 4B**). Thereafter, another third-generation EGFR-TKI aumolertinib (110 mg po qd) was administered to treat the NSCLC. The aumolertinib treatment was maintained with the cardiac failure treatment regimen for the next 4 months with routine examination. Later, tumor analysis revealed that the size of the tumor was partially decreased (2.9 × 1.6 cm) (**Figures 1D, I**). Fortunately, the cardiac function returned to normal (**Table 3**), as indicated by LVEF (57%) (**Figure 4A**) and the reduced level of NT pro-BNP (901 ng/L) (**Figure 4B**). Furthermore, the medicinal load was also reduced to furosemide (20 mg bid), spironolactone (20 mg bid), and aumolertinib (110 mg qd) for the next 4 months. Interestingly, regular follow-up showed gradual recovery in the symptoms (**Figures 1E, J**); tumor size reached stable disease (SD), the ejection fraction improved to 66% (**Figure 4A**), the pro-BNP decreased to 137 ng/L (**Figure 4B**), and the left ventricle function become normal, which showed significant improvement in the cardiac function as well as the NSCLC condition. There was a slight change in serological markers before and after switching to aumolertinib (**Table 2**), which indicated that the third-generation EGFR-TKIs performed excellently as anti-tumor medicines. The changes in electrocardiogram at cardiac failure and during recovery treatment were also monitored (**Figure 5**), which showed that RV5 was more than 2.5 mV when the patient suffered a cardiac failure, and the high voltage suggested left ventricular hypertrophy. The changes in myocardial enzymes at cardiac failure and during recovery treatment were monitored (**Table 2**), and the results showed abnormal levels of the lactic dehydrogenase (LDH) and creatine kinase of muscle and brain isozyme (CK-MB). This follow-up treatment that included alternative third-generation EGFR-TKI aumolertinib significantly soothed the condition of a patient with no further complaints of heart discomfort or liver and kidney function damage except fatigue.

**Table 2:** The changes in myocardial enzymes at cardiac failure and during recovery treatment.

Time	Myocardial Enzymes			
	AST	LDH	CK	CKMB
2020.04	11 U/L	161 U/L	20 U/L	15 U/L
2020.10	29.5 U/L	286 U/L	30.7 U/L	37 U/L
2020.11	19.7 U/L	284 U/L	39.1 U/L	31.6 U/L
2021.03	13.8 U/L	121 U/L	29 U/L	15.7 U/L
2021.07	9.6 U/L	127 U/L	21.3 U/L	15.7 U/L

**Table 3:** The left ventricular echocardiography data at cardiac failure and during recovery treatment.

Time	Left Ventricular Echocardiography Data					
	LVESD (mm)	LVEDD (mm)	LVESV (ml)	LVEDV (ml)	LVPWT (mm)	EF
2020.04	38	52	61	118	8	65%
2020.10	44	55	70	130	8	36%
2020.11	45	55	72	131	8	52%
2021.03	33	48	47	110	8	57%
2021.07	26	45	31	98	8	66%

LVESD, left ventricular end systolic diameter; LVEDD, left ventricular end diastolic diameter; LVESV, left ventricular end systolic volume; LVEDV, left ventricular end diastolic volume; LVPWT, left ventricular posterior wall thickness; EF, ejection fraction.

## Discussion

Lung cancer is one of the most common malignancies and the leading cause of cancer-associated deaths worldwide (11). About 15% of Caucasian and nearly 50% of Asian advanced NSCLC patients were detected with EGFR-positive mutation, mostly in exons 18–21 (12, 13). Uncontrolled activity of the EGFR can act as an active oncogenic driver and target for precision medicine intervention in lung cancer cells (14). Several drugs such as gefitinib, erlotinib, afatinib, and dacomitinib are being used for the treatment of patients with NSCLC who have EGFR mutations. However, over the past years, the tumors have ADR to chemotherapies specifically in patients with EGFR T790M mutation in exon 20 (15–17). Among available medicines, osimertinib is an FDA-approved third-generation EGFR inhibitor. This is highly selective for NSCLC patients with EGFR mutations or first- and second-generation TKI-resistant EGFR T790M mutations. The efficacy of osimertinib can be evaluated by a significant increase in the PFS (median: 18.9 vs. 10.2 months) and overall survival (median: 38.6 vs. 31.8 months) of patients with advanced EGFR-positive or T790M-positive NSCLC patients (18). In addition, osimertinib presented a safety profile consistent with previous reports; however, QT AE prolongation was 10% and serious AE (grade ≥3) prolongation was 1% (18, 19).

Among the AEs, cardiotoxicity is continuously rising when patients are treated with osimertinib. Several cases of heart failure related to osimertinib had been reported. For instance, Watanabe et al. reported a 78-year-old woman presenting with mild exertional dyspnea 3 weeks after starting osimertinib for the treatment of EGFR T790M-positive NSCLC, and she was diagnosed with congestive heart failure caused by the osimertinib (20). Oyakawa et al. reported an 84-year-old woman without any smoking history presenting with dilated and diffusely hypocontractile left ventricle (ejection fraction 33%) with minor pericardial effusion; osimertinib was discontinued, and furosemide, enalapril, and carvedilol were initiated (21). Okutucu et al. showed that a 64-year-old woman was diagnosed with metastatic lung adenocarcinoma harboring an EGFR mutation; 2 months after osimertinib initiation, which revealed that her left ventricular systolic function was depressed, with a globally reduced ejection fraction of 24%, they concluded that the heart failure had been caused by osimertinib inhibiting human epidermal growth factor receptor 2 (HER2) (22). Reale et al. reported 5 patients treated with osimertinib who suffered from heart failure; one patient died due to further worsening of cardiac condition, another patient was suspended due to reduction of EF, one patient received reduced osimertinib dosage, and the last 2 patients continued osimertinib after heart failure treatment. Osimertinib cardiotoxicity is more likely to be a type 2 cardiotoxicity and reversible (23). However, there is no evidence that resumption of osimertinib is harmful to patients after treating osimertinib-induced heart failure. The retrospective analyses of a pharmacovigilance database, the FDA AEs reporting system, reported a rare incidence (3.7%) regarding the occurrence of cardiotoxicity linked to EGFR-TKIs in 8,450 AEs. However, the results found that the reporting odds ratio for osimertinib compared with all other drugs was 5.4 for cardiac failure and 11.2 for QT prolongation, and compared with the 3 other EGFR TKIs, it was 2.2 for cardiac failure and 6.6 for QT prolongation (24). A systematic review and meta-analysis of randomized controlled trials (RCT) was undertaken to determine the incidence of osimertinib cardiac toxicities and showed that osimertinib notably increased the risk of cardiac toxicities with a risk ratio of 2.71 for cardiac failure and 2.62 for QT prolongation; thus, prompt monitoring and early intervention are warranted (25). Andrew et al. reported a 67-year-old woman treated with first-generation EGFR TKI erlotinib and then changed the osimertinib without resistance; 7 months after osimertinib initiation, she presented with shortness of breath and was diagnosed with heart failure and had to use erlotinib again. Osimertinib is not only highly specific for ErbB1 (EGFR), but also has demonstrated greater inhibition of wild-type ErbB2 (HER2) than that observed with erlotinib or afatinib. Whether this effect is responsible for increased cardiotoxicity requires further exploration (26). Kunimasa reported a retrospective, single-center study conducted in Japan. A total of 123 NSCLC patients with EGFR mutations were treated with osimertinib, and grade 3 or higher cardiac AEs were observed in 6 patients (4.9%). Five of those patients were women; their cardiac AEs included acute myocardial infarction (*n* = 1), heart failure with reduced LVEF (*n* = 3), and valvular heart disease (*n* = 2). For the patient described in case 1, LVEF recovery was delayed, and gefitinib was started 8 months after the AE; the patient in case 2 refused osimertinib re-administration and also received gefitinib. Osimertinib was re-administered to the patients described in cases 3 and 4, given the presence of the T790M mutation, whereas patients were treated with other EGFR-TKIs (cases 5 and 6). Altogether, osimertinib was discontinued in 4 of 6 patients and restarted in 2 patients; in one of those patients, osimertinib was re-administered at the reduced dose and the original dose in the other patient (27). Patel et al. reported that 3 patients suffered from cardiac failure after being initiated on osimertinib, 2 patients had basic heart disease, and LVEF decreased dramatically. Osimertinib was re-administered to only 1 patient, whereas the last 2 patients were treated with erlotinib (28). Shinomiya et al. reported a 76-year-old woman who underwent surgical resection of the left upper lobe plus lymph node dissection and used osimertinib as the second-line adjuvant therapy; 4 months after osimertinib initiation, cardiomyopathy secondary to osimertinib was diagnosed and osimertinib was discontinued (29). Ikebe et al. reported a rare case in which osimertinib simultaneously induced congestive heart failure and QT prolongation. The possible reason was a prior stereotactic thoracic radiotherapy; her heart was within the irradiation area, and radiation therapy could damage both cardiomyocytes and vasculature. She did not receive any other chemotherapy and died of cancer progression and cachexia at home 15 months after osimertinib discontinuation (30). In these case reports, most patients switched to other first-generation EGFR-TKIs because of osimertinib-induced cardiotoxicity, even if it was reversible.

With an increasing number of osimertinib-induced cardiotoxicity, the question arises, what could be the possible mechanism behind the cardiotoxicity triggered by osimertinib? EGFR is a receptor tyrosine kinase (RTK) in the erythroblasts leukemia viral oncogene homolog (ErbB)/human epidermal growth factor receptor (HER) family that includes HER2. HER2 is found to be important in cardiac development and the maintenance of normal cardiac structure and function under stress conditions. Moreover, treatment of cells with AZD9291 (osimertinib) inhibited phosphorylation of HER2 at moderate potency levels (31). Therefore, it is not unanticipated that cardiotoxicity has been observed with this agent.

Aumolertinib, a novel third-generation EGFR-TKI, has been used to treat NSCLC EGFR T790M-positive patients with progressive disease or recurrence as an alternative to other EGFR TKI therapies (32). Compared with osimertinib, the aumolertinib innovatively has an additional cyclopropyl group, which increases its anti-tumor activity, stability, and fat solubility. Like osimertinib, the aumolertinib also irreversibly binds with the EGFR T790M mutation sites and inhibits their function (32, 33).

Promising results have been observed for aumolertinib against lung cancer EGFR mutational cells, suggesting its specificity and admirable pharmacokinetic properties in mammals (34). Aumolertinib showed great tumor-regressing efficacy in an EGFR mutant brain metastasis model (35).

In phase II clinical APOLLO trials, aumolertinib has surprisingly shown significant performance with unique anti-tumor characteristics such as the primary endpoint, the overall response rate (ORR) was 68.9%, and the secondary endpoint, the median PFS of 12.4 months. In addition, the inhibitory effects of aumolertinib included the median duration of response (mDOR) of 15.1 months, and the disease control rate (DCR) of 93.4% (36). Additionally, the fat solubility of aumolertinib enables it to successfully cross the blood–brain barrier, thus showing adequate effectiveness in central nervous system (CNS) metastases: the CNS ORR was 60.9%, the CNS DCR was 91.3%, the median CNS PFS was 10.8 months, and the median CNS DOR was 12.5 months. It is worth mentioning here that PFS and OS of L858R and 19 deletion patients have similar benefits (36, 37). The most common treatment-related adverse reactions (TRAEs) ≥10% were blood creatine phosphokinase (CPK) increased (20.9%), rash (13.9%), aspartate aminotransferase (AST) increased (12.3%), white blood cell (WBC) count decreased (12.3%), alanine aminotransferase (ALT) increased (11.9%), and pruritus (10.7%); 15 (6.1%) patients had QT prolongation, and there was no ILD reported (36). The major AEs caused by aumolertinib include rash, diarrhea, fatigue, ALT/AST increase, and anemia, but none of the reported cases showed ILD and serious cardiotoxicity (36). Interestingly, the current patient had no history of any cardiac diseases, and the incidence of the cardiotoxicity induced by osimertinib was not obvious until aggravated after 6 months of continued treatment. Later, the treatment plan changed, which gradually improved the cardiac symptoms in 9 months.

In summary, we present an EGFR-positive NSCLC case with osimertinib-induced cardiac failure. Next, we switched the treatment plant to aumolertinib, which alienated the cardiac symptoms as well as tumorigenicity. Until now, the patient is being treated with aumolertinib for more than 9 months and maintained SD without any obvious AEs. The patient is regularly followed up to monitor the adverse reactions and improvement in treatment outcomes. Here, we report for the first time that switching from osimertinib to aumolertinib following the occurrence of osimertinib-induced cardiac failure was helpful in the gradual recovery of cardiac and NSCLC symptoms. It could be a potential therapeutic strategy for other patients with similar AEs.

## Data Availability Statement

The raw data supporting the conclusions of this article will be made available by the authors, without undue reservation.

## Ethics Statement

The studies involving human participants were reviewed and approved by the Ethics Committee of the Henan Provincial Peoples’ Hospital, China. The patients/participants provided their written informed consent to participate in this study.

## Author Contributions

QZ conceived the idea, collected data, and wrote the first draft of the manuscript. HL and JY performed data analysis, studied all clinical parameters of the patient, and reviewed and revised the manuscript. All authors contributed to the article and approved the submitted version.

## Conflict of Interest

The authors declare that the research was conducted in the absence of any commercial or financial relationships that could be construed as a potential conflict of interest.

## Publisher’s Note

All claims expressed in this article are solely those of the authors and do not necessarily represent those of their affiliated organizations, or those of the publisher, the editors and the reviewers. Any product that may be evaluated in this article, or claim that may be made by its manufacturer, is not guaranteed or endorsed by the publisher.
